# Emerging Trends in Health Promotion for People with Disabilities

**DOI:** 10.3390/ijerph15040742

**Published:** 2018-04-13

**Authors:** Brooks C. Wingo, James H. Rimmer

**Affiliations:** 1 Department of Occupational Therapy, University of Alabama at Birmingham (UAB), 1720 2nd Ave. S, Birmingham, AL 35294, USA; 2 UAB/Lakeshore Research Collaborative, Birmingham, AL 35294, USA; jrimmer@uab.edu

The need among people with disabilities to improve their own health and prevent/manage secondary conditions requires a better balance between reactive and anticipatory care. We are constantly reminded from reading the latest research that there is a great deal more people with disabilities can do to live a life of good health and well-being. However, for that to be the ‘rule’ rather than the ‘exception’, communities will need to become more inclusive by facilitating enabling environments that reduce barriers to participation. Unfortunately, entrenched socioeconomic disadvantages and structural, programmatic, and attitudinal barriers within the community are now widely recognized as major contributors to the health disparities and barriers to health promotion confronting people with disabilities. 

Public health programs and professionals who work in local and state health departments, schools, fitness and recreation centers, and health care facilities, must recognize the low rates of participation in health promotion reported among people with disabilities and begin to develop effective and cohesive strategies to address this problem. Recognizing this gap in programs and the health disparities associated with disability, this special issue addresses a number of key topics. 

A wide range of disabilities and intervention modalities are reported in this issue. In addition to physical disabilities such as spinal cord injury, a number of reports are also included that focus on individuals with intellectual disabilities (ID). Oviedo and colleagues report high levels of sedentary behavior among adults and older adults with ID, and older adults appear to accumulate even less activity than middle-adult counterparts [1]. Similarly, Hsieh presents results of an analysis on TV watching behaviors among adults with ID, and discusses correlates of TV watching behavior, as well as important potential protective factors that were inversely correlated with TV watching, including participation in organized sporting activities and involvement in education and employment activities [2]. A special emphasis is also given to providing support to parents and caregivers, who are an important and often understudied stakeholder group for promoting behavior change among people with disabilities [3,4,5].

Another key focus of this special issue is the importance of intervention implementation modalities. The growth of mobile technology and digital health resources presents a unique opportunity to reach segments of the population that are largely underserved and are typically excluded from health promotion interventions. In their article, “Mobile Healthcare and People with Disabilities: Current State and Future Needs”, Michael Jones and colleagues review current gaps in mobile health technology applicability for people with disabilities, and offer innovative future directions for research [6].

Finally, this special issue offers a series of articles focused on the importance of moving health promotion research past individual-level interventions into a knowledge translation framework that shapes policy and clinical care. Kerri Vanderbom discusses how the National Center on Health, Physical Activity and Disability (NCHPAD) is taking a systematic approach to promoting inclusive programming through communities, systems, and policies [7]. This call for a paradigm shift continues with the article, “Preparing Physical and Occupational Therapists to be Health Promotion Practitioners: A Call to Action”, in which David Morris and Gavin Jenkins discuss the importance of integrating health promotion into the skill sets of rehabilitation professions that have traditionally been grounded in a medical model [8].

The timeliness of this special issue could not have come at a better time. People with disabilities continue to lag behind the rest of the world’s population in major areas of health and health promotion access. Lack of health education and health awareness exacerbates the limited access to healthcare and healthcare follow-up, creating formidable barriers to effective health promotion for millions of people with disabilities across the world. We hope that this special issue will increase greater awareness among health professionals worldwide that people with disabilities need to be recognized as a major health disparity population who need and desire accessible and inclusive pathways for improving and maintaining their own health. 
